# Single-molecule imaging reveals dimerization/oligomerization of CXCR4 on plasma membrane closely related to its function

**DOI:** 10.1038/s41598-017-16802-7

**Published:** 2017-12-04

**Authors:** Baosheng Ge, Jun Lao, Jiqiang Li, Yao Chen, Yanzhuo Song, Fang Huang

**Affiliations:** State Key Laboratory of Heavy Oil Processing and Center for Bioengineering and Biotechnology, China University of Petroleum (East China), Qingdao, 266580 P. R. China

## Abstract

Dimerization and oligomerization of G-protein coupled receptors (GPCRs) have emerged as important characters during their trans-membrane signal transduction. However, until now the relationship between GPCR dimerization and their trans-membrane signal transduction function is still uncovered. Here, using pertussis toxin (PTX) to decouple the receptor from G protein complex and with single-molecule imaging, we show that in the presence of agonist, cells treated with PTX showed a decrease in the number of dimers and oligomers on the cell surface compared with untreated ones, which suggests that oligomeric status of CXCR4 could be significantly influenced by the decoupling of G protein complex during its signal transduction process. Moreover, with chlorpromazine (CPZ) to inhibit internalization of CXCR4, it was found that after SDF-1α stimulation, cells treated with CPZ showed more dimers and oligomers on the cell surface than untreated ones, which suggest that dimers and oligomers of CXCR4 tend to internalize more easily than monomers. Taken together, our results demonstrate that dimerization and oligomerization of CXCR4 is closely related with its G protein mediated pathway and β-arrestin mediated internalization process, and would play an important role in regulating its signal transduction functions.

## Introduction

G protein coupled receptors (GPCRs) form the largest and most diverse group of 7-trans-membrane proteins and play key roles in numerous processes *in vivo*, especially in trans-membrane signal transduction^1^. Based on their sequence homology, GPCRs are classically divided into three subfamilies: family A (Rhodopsin-like receptors), family B (secretin receptors) and family C (metabotropic glutamate receptors)^2^. It is reported that about 50% of the current therapeutic drugs under development target GPCRs. A better understanding of the structures and functions of GPCRs is therefore expected to facilitate greatly the rational design of drugs with increased efficacy and selectivity^3,4^.

Recently, dimerization and oligomerization have emerged as the main characteristics of GPCRs, and are believed to play an important role in regulating their signal transduction functions^5–7^. GPCRs from C family have been confirmed existing as homo- or hetero-dimer, or even higher oligomers on the cell surface^8,9^, and their dimeric structures are found necessary for them to move to the membrane, ligand activation as well as G protein binding, and thus are essential for realizing their functions^10^. However, the role of dimerization and oligomerization for some other GPCRs, such as those from family A and family B, is still highly controversial^10,11^. Many GPCRs from family A and B are found existing as dimers or oligomers on the cell surface^8,12–14^ and crystal structures were analysed^10,15^. However, both monomeric^16–18^ and dimeric status of GPCRs from family A and B^19^ have been shown to be active enough on their own for their signal transduction function. Until now, the relationship between GPCR dimerization and signal transduction function, and how receptor dimerization regulates their function are not well understood^8,10,20^.

CXCR4 belongs to family A GPCRs and is the natural receptor of stromal cell-derived growth factor (SDF-1α). It plays a key role in leukocyte trafficking, hematopoiesis, organ development and cancer metastases, as well as human immunodeficiency virus type 1(HIV-1) entry^21–23^. Thus it is an important therapeutic target for developing of efficient drugs. CXCR4 has been demonstrated to form constitutive homo- or hetero- dimers using fluorescence resonance energy transfer (FRET)^24,25^ and bioluminescence resonance energy transfer (BRET) methods^26^. It has also been reported that CXCR4 signaling can be influenced by receptor dimerization^24,27^ but the detailed relationship between oligomerization of CXCR4 and its function is yet unclear^8,20,28^.

Frequently, the activation of GPCR in the signal transduction process would be subjected to a variety of rigorous control or restrictions at different levels^1,29,30^. As trans-membrane proteins, GPCRs couple to an intracellular G protein complex in resting cells^31^. Upon extracellular ligand binding, the receptors are activated and undergo a rapid conformational rearrangement, then the G protein complex would be decoupled from the receptor to generate downstream responses^32^. Subsequently, the activated receptor promotes β-arrestin recruitment, which leads to receptor internalization. Ligands, GPCRs, G-proteins and β-arrestins typically show a high plasticity in engaging with different types of interaction partners^11^. With single-molecule techniques, we have shown that different agonists can effectively up-regulate or down-regulate the oligomeric status of CXCR4 on the cell surface, and binding ligands can realize their pivotal role by regulating the oligomeric status of CXCR4^33^. However, the more detailed relationship between CXCR4 dimerization, G protein complex coupling and β-arrestin mediated internalization, and whether the G protein mediated signal transduction and β-arrestin mediated internalization have an effect on the oligomeric status of the receptor, are so far unknown^6,29–31^.

In this paper, PTX is applied to decouple the CXCR4 receptor from its downstream G protein complex^11,34^. Using single-molecule imaging, we probe the influence of the decoupling of G protein complex on the oligomeric status of CXCR4 on the cell surface under the resting and SDF-1αactivated conditions. Moreover, with CPZ to prevent the formation of new clathrin-coated vesicles and then inhibit internalization of CXCR4^11,35^, the relationship between receptor internalization and oligomeric status of CXCR4 on the cell surface are also explored. Our results provide more detailed information on the relationship of oligomerization of CXCR4 with its signal transduction function, and would benefit for a better understanding of the mechanism of GPCR signal transduction and related effective drug design.

## Results

### Effect of G protein decoupling on the oligomeric status of CXCR4-EGFP on resting cells

PTX is one of the G-protein dependent signaling inhibitors resulting from loss of coupling of G protein complex and receptors^34,36^. To study the relationship between the oligomeric status of CXCR4 and G-protein mediated signal transduction, cells stably transfected with CXCR4-EGFP were treated with 200 ng/ml PTX to decouple G protein complex from CXCR4 receptors^11^. Chemotaxis experiments confirmed that after PTX treatment the stably transfected cells lost their chemotactic activity upon SDF-1α stimulation (Fig. S1), which implied that G protein complexes have been successfully decoupled from CXCR4. Dot-blotting and confocal imaging analysis demonstrated that this PTX treatment does not affect the expression level and membrane distribution of CXCR4-EGFP (Figs S2 and S3), which is suitable for following single-molecule total internal reflection fluorescence microscopy (TIRFM) analysis.

**Figure 1:**
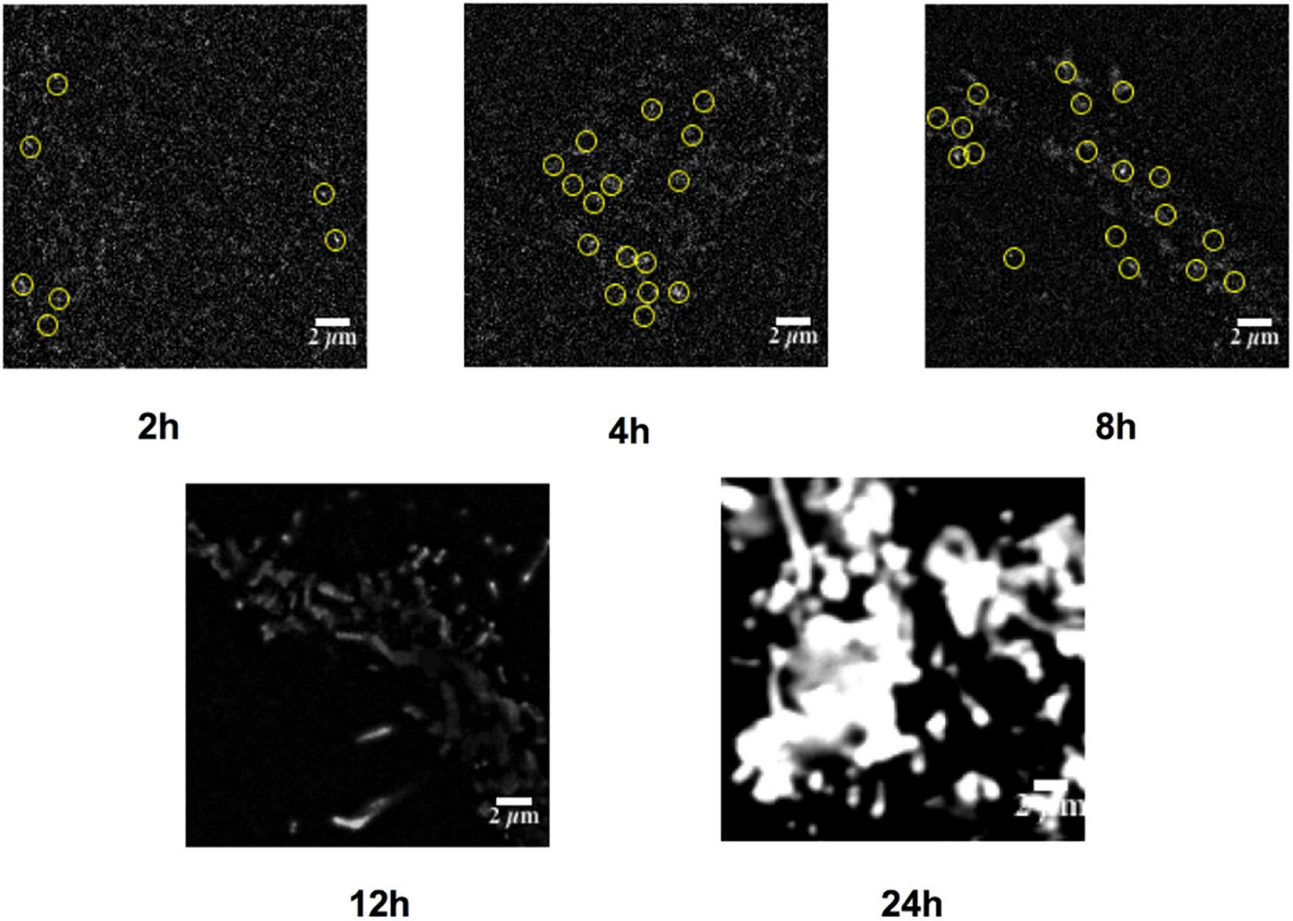
Single-molecule imaging of PTX treated cells at different induction times. Stable cells at induction time of 2, 4, 8, 12 and 24 h were imaged with TIRFM. The image is a section (20 × 20 μm) of the first frame from a stack of images (Movie S1) with background subtracted. The diffraction-limited spots (5 × 5 pixel regions, 800 × 800 nm) enclosed with yellow circles represented the signals from individual CXCR4-EGFP molecules, and was chosen for intensity analysis (scale bar = 2 µm).

**Figure 2:**
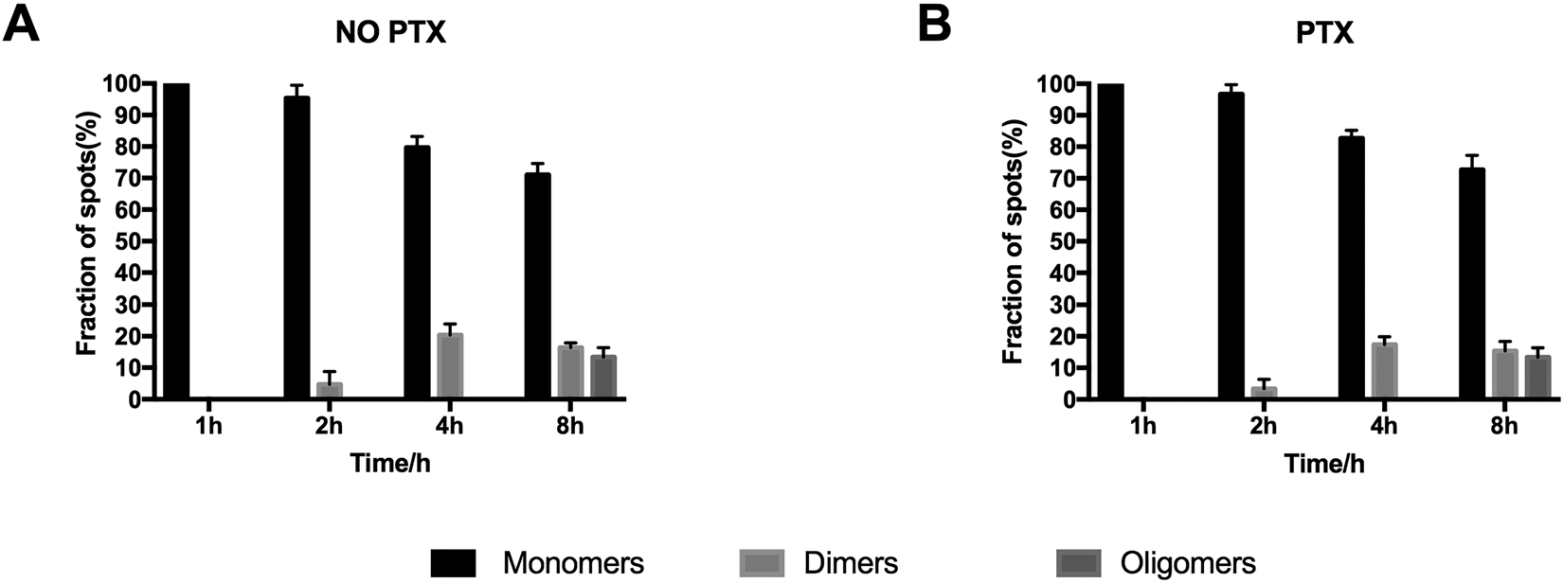
Oligomeric status of CXCR4 on the cell surface revealed by single-molecule imaging. The experiments were carried out using the stably transfected T-Rex-HEK293 cells under resting state (without SDF-1α stimulation) and cells at different induction times were characterized respectively. (**A**) Relative amount of monomer, dimer and oligomer of CXCR4-EGFP on the cell surface at induction times of 1, 2, 4, and 8 h without PTX treatment; (**B**) Relative amount of monomer, dimer and oligomer of CXCR4-EGFP on the cell surface at induction times of 1, 2, 4, and 8 h with PTX treatment.

We have previously shown that the expression level of CXCR4 can significantly influence their oligomeric status on cell surface^33^. Therefore, after PTX treatment to decouple G protein complex from receptors, the oligomeric status of CXCR4 on the cell surface was characterized at different induction times of 1, 2, 4, 8, 12 and 24 h using single-molecule imaging. Fluorescence spots extracted from single-molecule images were then extracted and their photobleaching steps were analyzed to discriminate monomers, dimers and oligomers according to our previous reports (Movie S1 and Fig. S4)^33^. As shown in Fig. 1, at the initial stage (especially with 1 and 2 h induction time), the expression level of CXCR4 on the PTX treated cell surface was very low and CXCR4 existed mainly as monomers. With increasing induction time, the expression level of CXCR4 also increased. Dimers began to appear for up to 2 h induction time and oligomers appeared at 8 h. We then analyzed the oligomeric status of CXCR4-EGFP proteins on the cell membrane with and without PTX treatment using photobleaching method. As a result, the oligomeric status of CXCR4-EGFP treated by PTX at different induction times (1, 2, 4 and 8 h) was found to be similar to that of the untreated one (Fig. 2), and there is no significant difference according to Mann-Whiney *t*-test (P < 0.01), which suggests that the decoupling of G protein complex has little effect on the oligomeric status of CXCR4 on resting cells.

### Effect of G protein decoupling on the oligomeric status of CXCR4 upon activation

**Figure 3:**
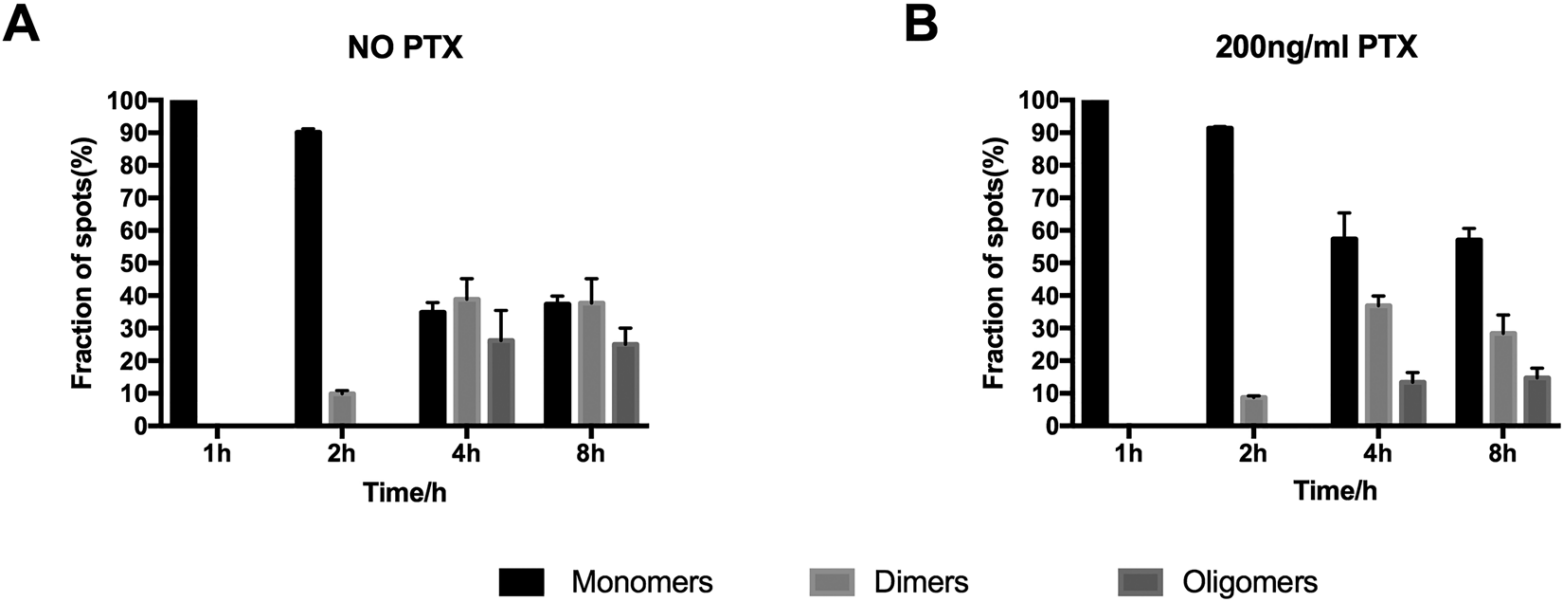
Oligomeric status of CXCR4 on the cell surface stimulated by SDF-1α. The experiments were carried out using the stably transfected T-Rex-HEK293 cells at different induction times with single-molecule imaging. (**A**) Relative amount of monomer, dimer and oligomer of CXCR4-EGFP on the cell surface at induction times of 1, 2, 4, and 8 h without PTX treatment; (**B**) Relative amount of monomer, dimer and oligomer of CXCR4-EGFP on the cell surface at induction times of 1, 2, 4, and 8 h with PTX treatment.

In order to characterize the influence of G protein complex decoupling on the oligomeric status of CXCR4 under activated conditions, cells were pre-cultured in DMEM medium supplemented with 200 ng/ml PTX for 12 h, and then induced by 1 μg/ml tetracycline for 1, 2 and 4 h. After that, 1 μM SDF-1α was added and incubated for 10 min, and then applied for single-molecule detection. The cells without PTX treatment were employed as controls. Figure 3 shows that there is no obvious difference between the oligomeric status of CXCR4 with and without PTX treatment at induction times of 1 h and 2 h. However, with an increase of the expression level of CXCR4 after 4 h of induction, the ratios of monomer, dimer and oligomer of CXCR4 in PTX treated cells were significantly different from those of untreated cells (Fig. 3). In addition, CXCR4 in PTX treated cells showed fewer dimers and oligomers than the untreated group, which indicates that upon activation by SDF-1α, the oligomerization of CXCR4 on cell surface could be significant influenced by the decoupling of G protein complex. This means that G-protein coupling and oligomerization of CXCR4 are closely related to the events during the CXCR4 signal transduction process.

### Effect of internalization on the oligomeric status of CXCR4-EGFP on resting cells

Internalization is another important way for regulation of GPCR activation and signaling strength^23,37^. Upon agonist stimulation, GPCRs are activated and then recruit β-arrestin from cytoplasm to membrane, which in turn links to the AP2 adaptor complex and facilitates their clathrin dependent endocytosis for desensitization or regeneration^11^. Chlorpromazine (CPZ), as a water-soluble inhibitor, can cause loss of clathrin from the cell surface in the coated pits, which then prevents formation of new clathrin-coated vesicles (CCVs) and inhibits internalization of GPCRs induced by agonist stimulation^11,38^.

Confocal microscopy imaging showed that after CPZ treatment, fluorescence spots of CXCR4-EGFP were found to be distributed mainly on the cell membrane after SDF-1α stimulation, and no obvious internalization was observed. In contrast, for the CPZ untreated cells, internalization was not inhibited, and fluorescence spots of CXCR4-EGFP were found to be distributed mainly in the cytoplasm with few on the cell surface (Fig. S5), which indicated that a large-scale internalization had been induced. These results clearly showed that the internalization of CXCR4-EGFP stimulated with SDF-1α could be effectively inhibited by CPZ treatment.

**Figure 4:**
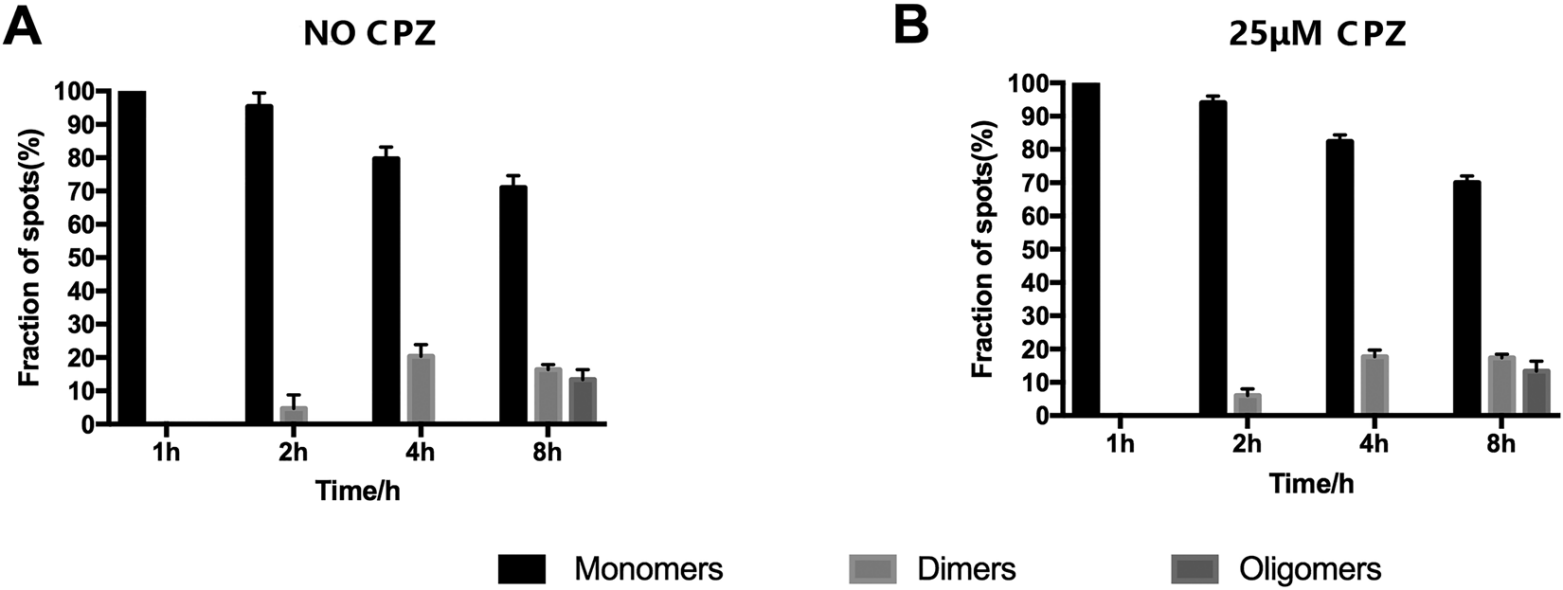
Oligomeric status of CXCR4 on the cell surface treated and untreated by CPZ. The experiments were carried out using the stably transfected T-Rex-HEK293 cells under resting state (without SDF-1α stimulation) and cells at different induction times were characterized by single-molecule imaging respectively. (**A**) Relative amount of monomer, dimer and oligomer of CXCR4-EGFP on the cell surface at induction times of 1, 2, 4, and 8 h without CPZ treatment; (**B**) Relative amount of monomer, dimer and oligomer of CXCR4-EGFP on the cell surface at induction times of 1, 2, 4, and 8 h with CPZ treatment.

To determine whether CPZ treatment affects the oligomeric status of CXCR4-EGFP on cell surface, the cells described above at induction times of 1, 2, 4, 8, 12 and 24 h were divided into two groups, one treated with 25 μM CPZ in DMEM medium and the other containing no CPZ. After being incubated at 37 °C for 30 min, cells were changed into DMEM medium containing 10% FBS-free without phenol red, and then imaged using the TIRFM method. As shown in Fig. 4, the oligomeric status of CXCR4 in CPZ treated cells showed no significant difference (P < 0.01 in Mann-Whiney *t*-test) from the untreated group at different induction times, indicating that CPZ treatment had no significant effect on the oligomeric status of CXCR4 at resting conditions.

### Effect of internalization on the oligomeric status of CXCR4-EGFP upon activation

**Figure 5:**
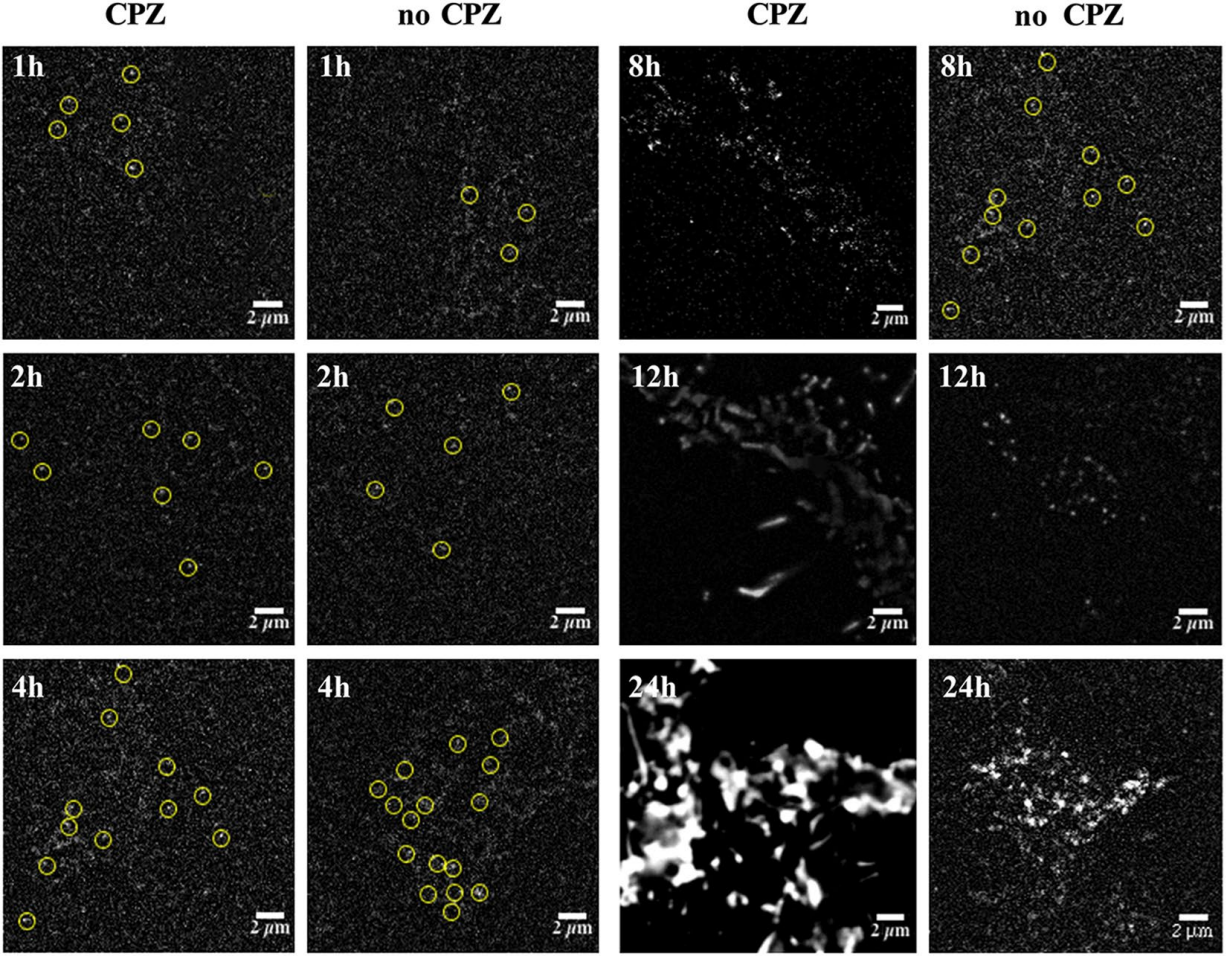
Single-molecule imaging of stably transfected cells with or without CPZ treatment at different induction times. Stably transfected cells at induction time of 1, 2, 4, 8, 12 and 24 h were imaged with TIRFM. The image is a section (20 × 20 μm) of the first frame from a stack of images (Movie S2) with background subtracted. The diffraction-limited spots (5 × 5 pixel regions, 800 × 800 nm) enclosed with yellow circles represented the signals from individual CXCR4-EGFP molecules, and was chosen for intensity analysis (scale bar = 2 µm).

**Figure 6:**
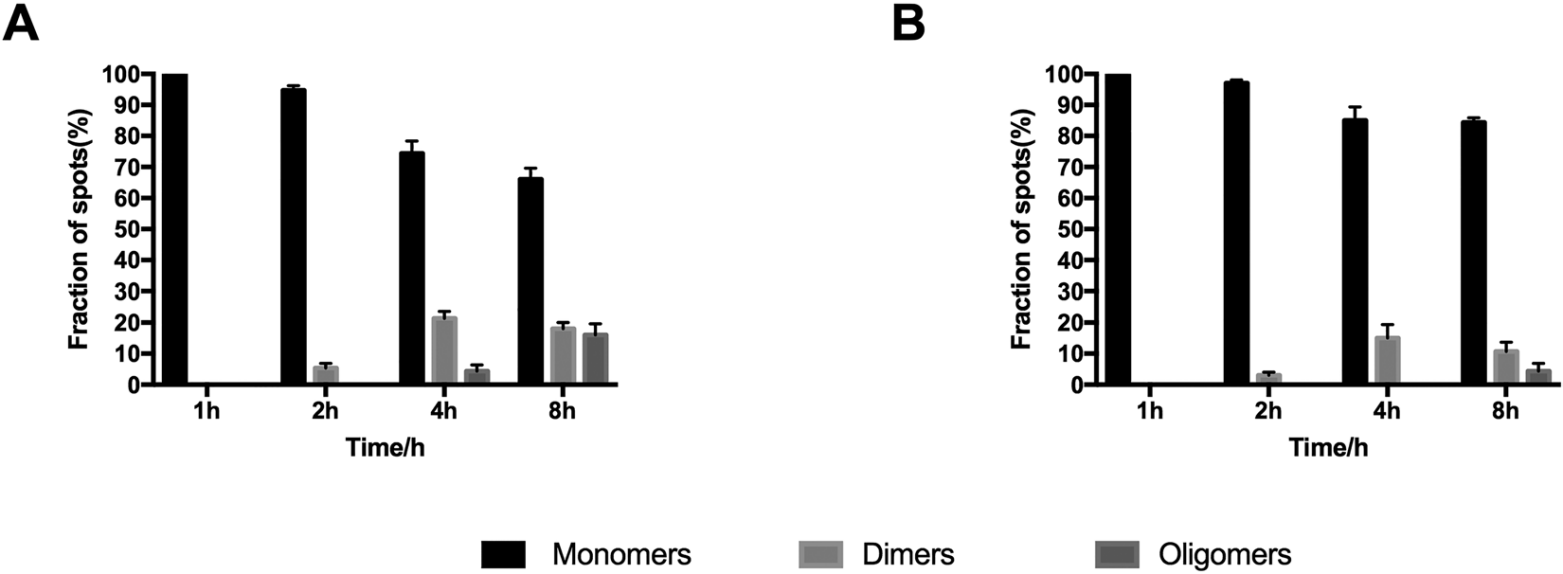
Oligomeric status of CXCR4 on the CPZ treated cell surface stimulated with SDF-1α. The experiments were carried out using the stably transfected T-Rex-HEK293 cells and characterized at different induction times by single-molecule imaging respectively. (**A**) Relative amount of monomer, dimer and oligomer of CXCR4-EGFP on the cell surface at induction times of 1, 2, 4, and 8 h with CPZ treatment; (**B**) Relative amount of monomer, dimer and oligomer of CXCR4-EGFP on the cell surface at induction times of 1, 2, 4, and 8 h without CPZ treatment.

In order to detect the effect of internalization on the oligomeric status of CXCR4-EGFP under activated conditions, the stably transfected cells were first treated with 25 μM CPZ at 37 °C for 30 min, stimulated with 100 nM SDF-1α for another 30 min, and then subjected to single-molecule imaging. As shown in Fig. 5, at induction time of 1–4 h, the expression level of CXCR4-EGFP was very low, and the oligomeric status of CXCR4 in CPZ treated cells was not significantly different from the untreated group. When the induction time was prolonged as 4–8 h, the expression level of CXCR4-EGFP increased similarly to native tumor cells^33^. Since the internalization of CXCR4 was inhibited in CPZ treated cells, upon SDF-1α stimulation, there was clearly oligomerization of CXCR4 on the CPZ treated cell membrane. However, for the CPZ untreated cells, internalization was not inhibited. Therefore, after SDF-1α stimulation, most receptors underwent internalization, and the total amount of CXCR4-EGFP on the cell membrane was reduced significantly, and less oligomer could be detected than those on the CPZ treated cells (Fig. 6). For the cells with induction times of 12 and 24 h, the concentration of CXCR4 was too high to be studied with single-molecule fluorescence techniques. Compared with the CPZ treated cells, most large aggregates and oligomers of CXCR4 in the untreated cells have been internalized after SDF-1α stimulation, just with some smaller oligomers and monomers remaining on the cell surface (Figs 5 and 6). These results suggest that internalization has a significant effect on the oligomeric status of CXCR4 on cell surface, and large aggregates and oligomers of CXCR4-EGFP may be more likely to undergo internalization than dimers and monomers.

## Discussion

GPCRs had been considered as functional monomeric units for a long period, but recently more and more GPCRs have been reported existing as dimers or even oligomers^5^. It is not yet clear why GPCRs dimerize and what the relationship between dimerization and their signal transduction function is^8,10^. Since dimerization of GPCRs is supposed to serve as an effective way to regulate their signal transduction function, therefore the relationship between dimerization of GPCRs and their signal transduction function has attracted much interest^20,23,39^. Shedding more lights on this issue is believed to facilitate greatly rational design of drugs with increased efficacy and selectivity^29,32,40^.

Due to the promiscuity of GPCRs, the same receptor can bind with various agonists, and vice versa, which then induce different responses^11^. To fulfill their functions, the signal transduction processes of GPCRs are believed to be subjected to a variety of strict control at different level. Using single-molecule technique, we have shown that different agonists can regulate the oligomeric level of CXCR4 on cell surface^33^, which suggests that agonists can realize their pivotal role by regulating the oligomeric status of CXCR4. However, the detailed relationship between the oligomeric status of CXCR4 and G-protein coupling and β-arrestin mediated internalization are unclear so far^23,31,32^.

To answer this question, PTX was used to promote the ADP-ribosylation of Gα protein to prevent GDP from replacing by GTP, which leads to the decoupling of G protein complexes from GPCRs^11,36^. This PTX treatment does not influence the expression level, insertion and distribution of CXCR4 on cell surface, which is suitable for us to explore the relationship between CXCR4 oligomerization and their signal transduction function using single-molecule imaging. As a result, we found that the oligomeric status of CXCR4 in PTX treated cells without agonist stimulation showed no obvious difference with untreated one at different induction times, which suggested that the decoupling of G protein complex has no significant impact on oligomerization of CXCR4 at resting conditions. However, once activated by agonists, significant difference of oligomeric status of CXCR4 were found between PTX treated and untreated cells, and PTX treated cells showed less dimers and oligomers than untreated one, which indicates that the coupling of G protein complex as well as ligand binding could induce oligomerization of CXCR4 on cell surface, and more oligomers and dimers would be needed for CXCR4 to be activated to decouple the G protein complex. These results suggested that the oligomerization of CXCR4 could be an important regulation way during G-protein mediated signal transduction of CXCR4^25^.

Internalization has been deemed as another important regulation way during signal transduction of GPCRs^41^. After being activated by agonists, most GPCRs proceed to be phosphorylated, induce recruitment of β-arrestin and the AP2 adaptor complex, and then facilitate their clathrin dependent internalization for desensitization. Results based on FRET analysis reported that both monomers and dimers of family A GPCRs, such as β_2_-AR, could bind with their agonists, be activated and then internalize respectively^42^. This FRET measurement only provides some information on FRET efficiency of β_2_-AR on cell surface and cytoplasm, but cannot distinguish the difference of how monomers, dimers and oligomers internalize respectively^33^. Using single-molecule techniques, we show that upon activated with SDF-1α, the oligomeric status of CXCR4-EGFP on CPZ treated cells are completely different from cells not treated with CPZ. Because the internalization has been inhibited^11,36^, large oligomers were found distributed mainly on the surface of CPZ treated cells with the increase of induction time. However, for cells not treated by CPZ, because of internalization, large aggregates and oligomers of CXCR4 on cell surface were much less than those of CPZ treated cells, and monomers and dimers were dominant. These results suggested that aggregates and large oligomers of CXCR4-EGFP would be more easily to internalize into cytoplasm than monomers and dimers. It should be noted that our results are still insufficient to determine whether the monomer is internalized after stimulation, thus it should not be excluded that this decrease of aggregates and large oligomers of CXCR4 are caused by reduction of CXCR4-EGFP concentration on cell membrane because of internalization.

**Figure 7:**
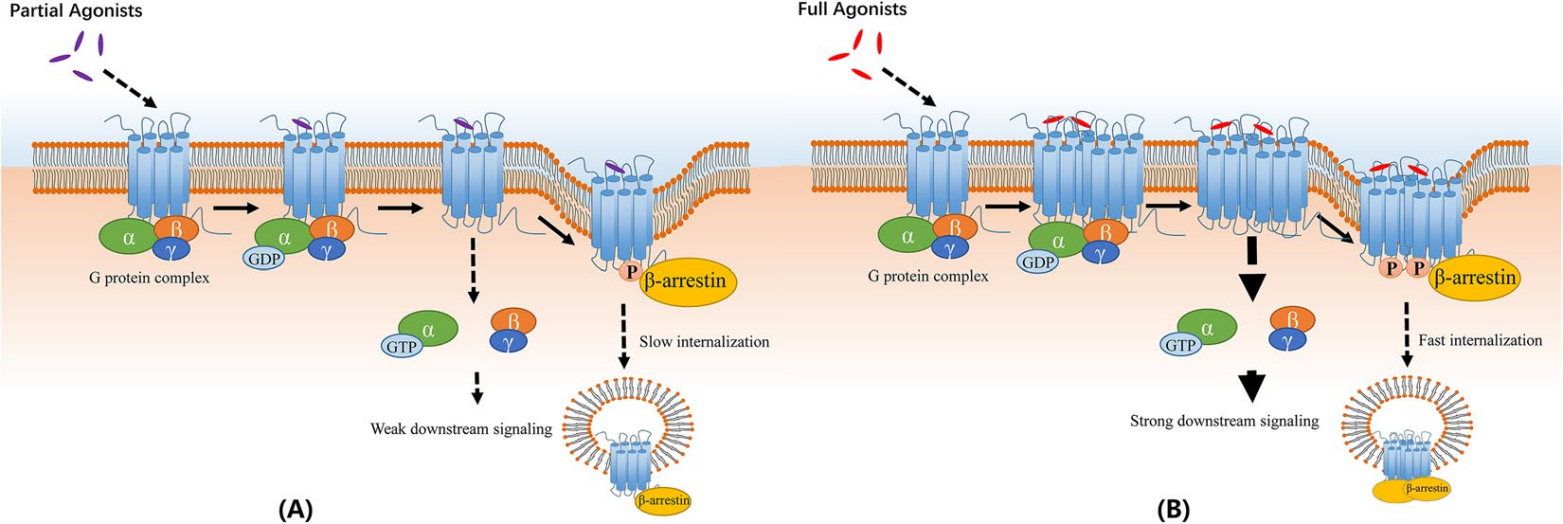
Schematic diagram for relationship of GPCR dimerization and their trans-membrane signal transduction functions. (**A**) Schematic diagram for signal tranduction of GPCRs activated with partial agonists; (**B**) Schematic diagram for signal tranduction of GPCRs activated with full agonists.

According to our previous reports^33^, full agonists can induce significantly increasing the number of dimer or oligomers of CXCR4, and partial agonists show slight influence on dimerization of CXCR4. In this study, we showed that less oligomers and dimers were found for PTX treated cells than untreated one, which means that more oligomers and dimers were needed for CXCR4 to be activated to decouple the G protein complex. And aggregates and large oligomers of CXCR4-EGFP would be more easily to internalize into cytoplasm than monomers and dimers. Taken together, we then propose that full agonists can bind with CXCR4 monomers or dimers, and induce dimerization/oligomerization of receptors, which can generate fully activated downstream signal (G-proteins mediated pathway), and then these dimers of CXCR4 can easily be internalized for desensitization (Fig. 7). While partial agonists can bind with CXCR4 monomers, but cannot induce dimerization effectively, and then just partly activate the G protein complex and generate a weak downstream signaling. These proposed models need to be demonstrated with more following studies on dimerization of family A GPCRs based on single-molecule techniques.

## Conclusions

Using signaling inhibitors to inhibit downstream signaling pathway of CXCR4, the relationship of CXCR4 dimerization/oligomerization with its trans-membrane signal transduction function were characterized with single-molecule techniques. It was found that the decoupling of G protein complex has little effect on the oligomeric status of CXCR4 on resting cells. However, upon activated by SDF-1α, the decoupling of CXCR4 from G protein complex shows fewer dimers and oligomers than untreated ones, which suggests that dimerization of CXCR4 is closely related with the decoupling of G protein complex during the signal transduction process. Moreover, with chlorpromazine (CPZ) to inhibit the internalization of CXCR4, we found that the oligomeric status of CXCR4 can also be significantly influenced by the β-arrestin mediated internalization, and dimers and oligomers of CXCR4 tend to internalize more easily than monomers. Our results demonstrate that the dimerization and oligomerization of CXCR4 are closely related to its functions, and may play key roles in the regulation of this important trans-membrane signal transduction process.

## Materials and Methods

### Cell culture

T-REx-293 cells stably transfected with CXCR4-EGFP plasmids were constructed according to our previous report^33^ and cultured in Dulbecco’s Modified Eagle Medium (DMEM, Hyclone, USA) supplemented with 10% fetal bovine serum (FBS, Sijiqing, China) at 37 °C and 5% CO_2_. To observe CXCR4-EGFP molecules, cells were first induced by 1 μg/ml tetracycline. After being washed with PBS (137 mM NaCl, 2.7 mM KCl, 10 mM Na_2_HPO_4_, 2 mM KH_2_PO_4_, pH 7.4), cells were directly imaged in DMEM without phenol red using total internal reflection fluorescence microscopy (TIRFM). For the SDF-1α (Prospec, USA) stimulation experiments, cells were incubated with 1 μM SDF-1α in the DMEM without FBS for 10 min at 4 °C before imaging, washed twice with PBS, and then imaged under TIRFM. To exclude the interference of protein density on photobleaching analysis, stably transfected cells at different expression times, typically 1 h, 2 h, 4 h, 8 h, 12 h and 24 h, were also characterized.

### PTX treatment to decouple the G protein complex from CXCR4

PTX (Sigma, USA) was used to detach G protein complex from CXCR4 in living cells^34^. Cells stably transfected with CXCR4-EGFP were pre-incubated in DMEM medium containing 200 ng/ml PTX for 12 h, and then induced by 1 μg/ml tetracycline in the presence of 200 ng/ml PTX. The influences of PTX treatment on expression level and membrane insertion of CXCR4 at different induction times were also characterized. For SDF-1α stimulated experiments, 1 μM SDF-1α was added and incubated for 10 min, and then applied for single-molecule detection.

### CPZ treatment to inhibit the internalization of CXCR4

For CPZ treatment, cells at different induction times were incubated in regular medium containing 25 μM CPZ (Sigma, USA) for 30 min, and then 100 nM SDF-1α was added and incubated for 30 min to stimulate receptors for internalization. Prior to imaging, the medium was replaced by phenol red-free DMEM and single-molecule or confocal imaging was done in the absence or presence of 100 nM SDF-1α.

### Single-molecule fluorescence imaging and analysis

Single-molecule fluorescence imaging was performed as previously reported^33^ on an inverted Nikon Ti series microscope, which equipped with a total internal reflective fluorescence illuminator, a 100×/1.49 NA Plan Apochromat TIR objective, and an intensified electron-multiplying charge-coupled device (EMCCD) camera (DU897; Andor). Briefly, EGFP-CXCR4 on the cell surface was excited at 488 nm using a 7 mW solid-state laser (Cobolt MLD 488 nm), and the collected fluorescent signals were passed through two filters, B-2A cubes BA510IF and HQ 525/50 (Nikon, Japan), before being directed onto the EMCCD camera. The gain of the EMCCD camera was set as 296. Movies of 100–500 frames were acquired for each sample, at a frame rate of 10 Hz.

For single-molecule imaging analysis, the rolling ball method in ImageJ software (National Institute of Health) was employed for the background subtraction from each frame. Fluorescent spots (region of interest) were selected from the first frame of each movie, and then analyzed using Speckel Trackerj software.

### Dot-blot analysis

Dot-blot analysis was carried out to estimate quantitatively the expression level of CXCR4 with or without PTX treatment after induction^43^. For PTX treatment, cells were first incubated in DMEM medium containing 200 ng/ml PTX for 12 h, and then cells with different induction times were lysed with PBS buffer containing 2% (w/v) Fos-choline 14 (Anatrace, USA) and 1 mM PMSF for 1 h at 4 °C. After being centrifuged at 13,000 g for 10 min, the supernatant was collected, 3 μL of each sample was picked up and dotted onto the nitrocellulose membrane. The membrane was air dried for 20 min and then blocked using 5% (w/v) milk. The membrane was then incubated with mouse anti-CXCR4 monoclonal antibody (Abcam, USA) for 1 h. After washing 5 times, HRP-labeled goat anti-mouse secondary antibody (Tiangen, China) was then added and incubated for another 1 h. Finally, the membrane was developed with SuperSignalWest Pico reagent and imaged on a FLA-5100 imaging system (Fuji, Japan). The intensity of the dot-blot was analyzed by MultiGauge Ver.3.X software.

### Chemotaxis tests

Chemotaxis tests of Hela and stably transfected T-REx-293 cells with or without PTX treatment were carried out using Transwell chambers (Corning, USA) respectively^33^. Cells were first divided into two groups, one treated by 200 ng/ml PTX for 12 h, and the other group not. After induction, cells were re-suspended in DMEM containing 0.5% (v/v) bovine serum albumin. Portions (100 μl) of the cell suspension were placed in the upper wells of Transwell chambers (500 μl), containing a bare filter with a pore size of 8 μm. The same medium (500 μl) containing 100 nM SDF-1α was placed in the lower chambers. After incubation for 4 h at 37 °C in a moist atmosphere containing 5% CO_2_, the cells that migrated through the filter were stained with crystal violet and counted with ImageJ.

### Ethical Statement

This article does not contain any studies with human participants or animals performed by any of the authors.

### Impotance statement

Dimerization and oligomerization of G-protein coupled receptors (GPCRs) have emerged as important characters during their trans-membrane signal transduction. However, until now the relationship between GPCR dimerization/oligomerization and their signal transduction function is still uncovered. Here, using pertussis toxin (PTX) and chlorpromazine (CPZ) to decouple CXCR4 from G protein complex and inhibit internalization of CXCR4 respectively, we explore the relationship between dimerization of CXCR4 and its signal transduction functions using single-molecule imaging techniques. Our results demonstrated that the oligomeric status of CXCR4 on the cell surface is closely related to its G protein mediated pathway and internalization process. To the best of our knowledge, these results constitute the first report of the relationship between CXCR4 dimerization and its signal transduction function, which provide important information for better understanding of the mechanism of trans-membrane signal transduction and benefit for further efficient drug design.

## Electronic supplementary material

Supporting Information

Movie S1
