# Supplementary material for: Single-molecule imaging reveals dimerization/oligomerization of CXCR4 on plasma membrane closely related to its function
Source: Sci Rep. 2017 Dec 4;7:16873. doi: 10.1038/s41598-017-16802-7 (PMC5715067; doi:10.1038/s41598-017-16802-7)
Supplement: Supplementary file 1 — Supporting Information [file 41598_2017_16802_MOESM1_ESM.doc]

**Supplementary Information**

Single-molecule imaging reveals dimerization/oligomerization of CXCR4 on plasma membrane closely related to its function

Baosheng Ge^†*^, Jun Lao^†^, Jiqiang Li, Yao Chen, Yanzhuo Song, Fang Huang*

*State Key* *Laboratory of Heavy Oil Processing and Center for Bioengineering and Biotechnology, China University of Petroleum (East China), Qingdao 266580, P. R. China*

^†^These authors contributed equally.

^*^To whom correspondence may be addressed:

BG: gebaosheng @upc.edu.cn; Tel: 0086-532-86981135, Fax: 0086-532-86981135

FH: fhuang@upc.edu.cn Tel: 0086-532-86981560, Fax: 0086-532-86981560


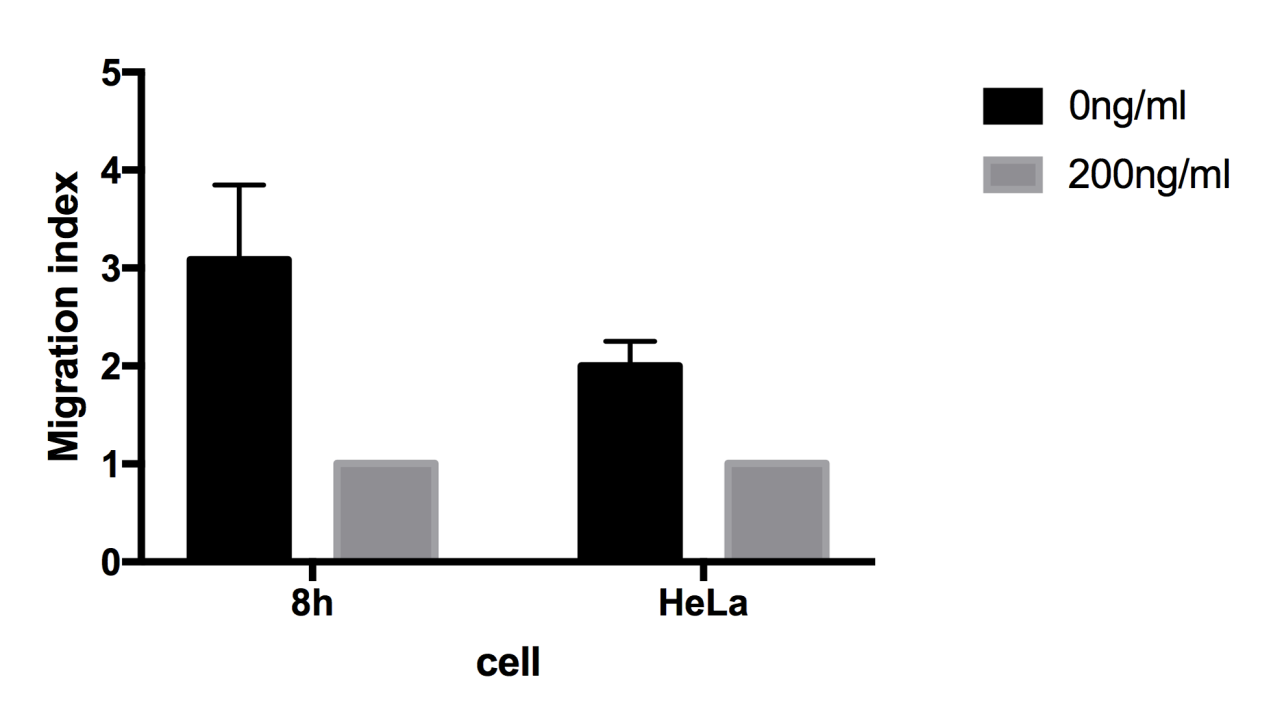


**Fig. S1 Chemotactic index of Hela and stably transfected cells with and without PTX treatment.**

**
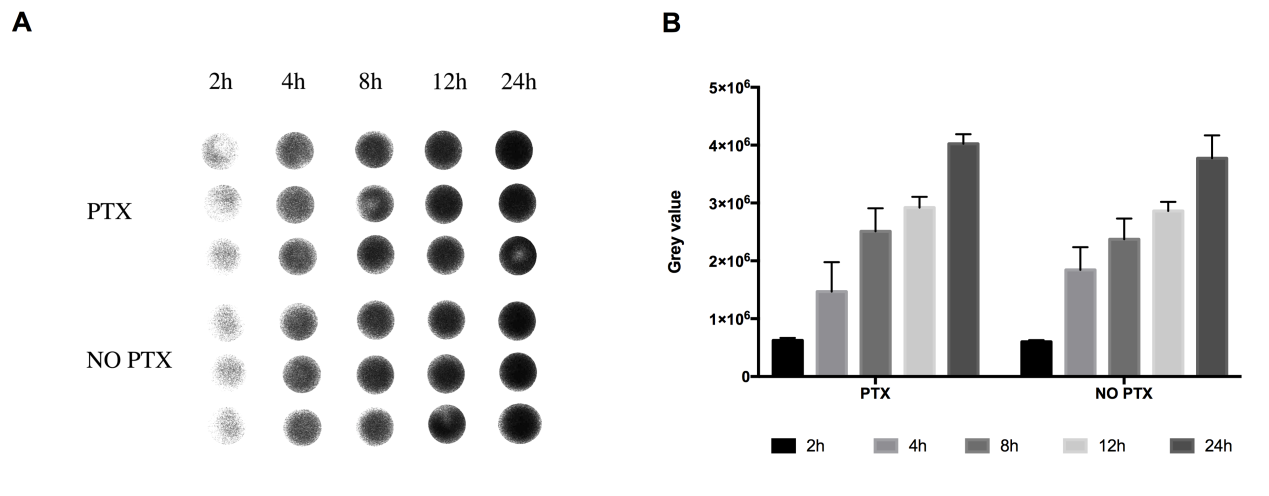
**

**Fig. S2 Effect of PTX treatment on expression level of CXCR4 in living cells using dot blot analysis. (**A) Dot blot image of CXCR4 in cells with and without PTX treatment at different induction times. (B) Dot blot intensities of CXCR4 in cells with and without PTX treatment at different induction times.


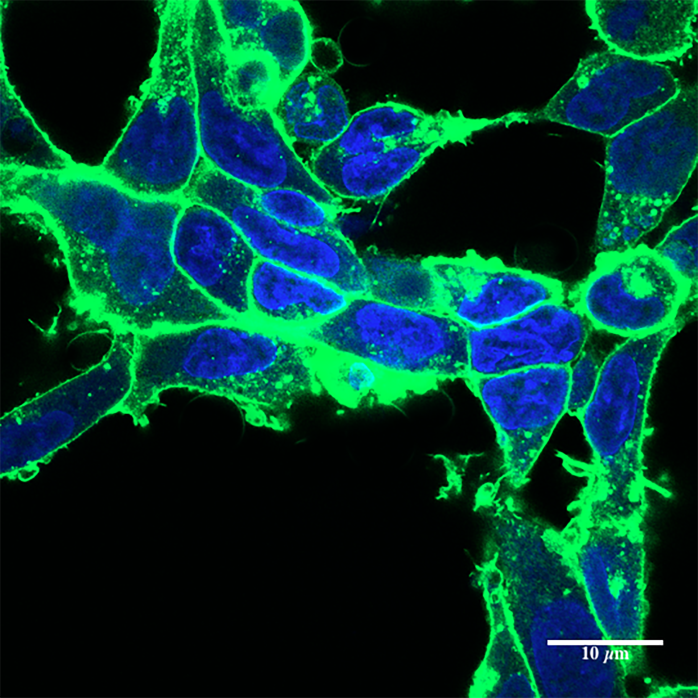


Fig. S3 The imaging of stably transfected T-Rex-HEK293 cells treated by PTX under laser confocal microcopy after 24 h induction. Green and blue represent CXCR4-EGFP and the Hoechst 33258-stained nucleus, respectively.


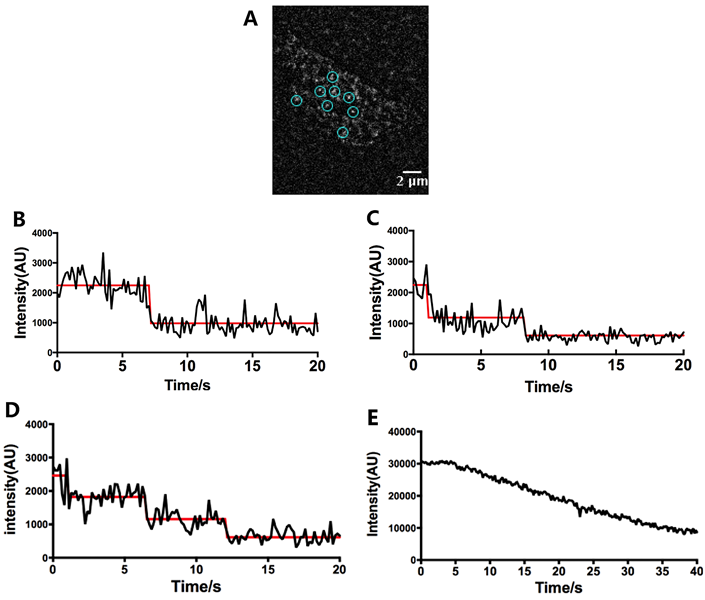


**Fig. S4 Single-molecule image of CXCR4-EGFP molecules in stably transfected T-REx-293 cells. (A)** A typical single-molecule image of CXCR4-EGFP on the living cell surface. Stable cells after 4 h Tetracycline induction were imaged with TIRFM. The image is a section (20×20μm) of the first frame from a stack of images (Movie S1) with background subtracted. The diffraction-limited spots(5×5 pixel regions，800×800nm) enclosed with cyan circles represented the signals from individual CXCR4-EGFP molecules, and was chosen for intensity analysis. (B) One representative time course of CXCR4-EGFP emission after background correction showed one-step bleaching. (C) One representative time course of CXCR4-EGFP emission after background correction showed two-step bleaching. (D) One representative time course of CXCR4-EGFP emission after background correction showed three-step bleaching. (E) One representative time course of CXCR4-EGFP emission after background correction showed multiple-step bleaching.


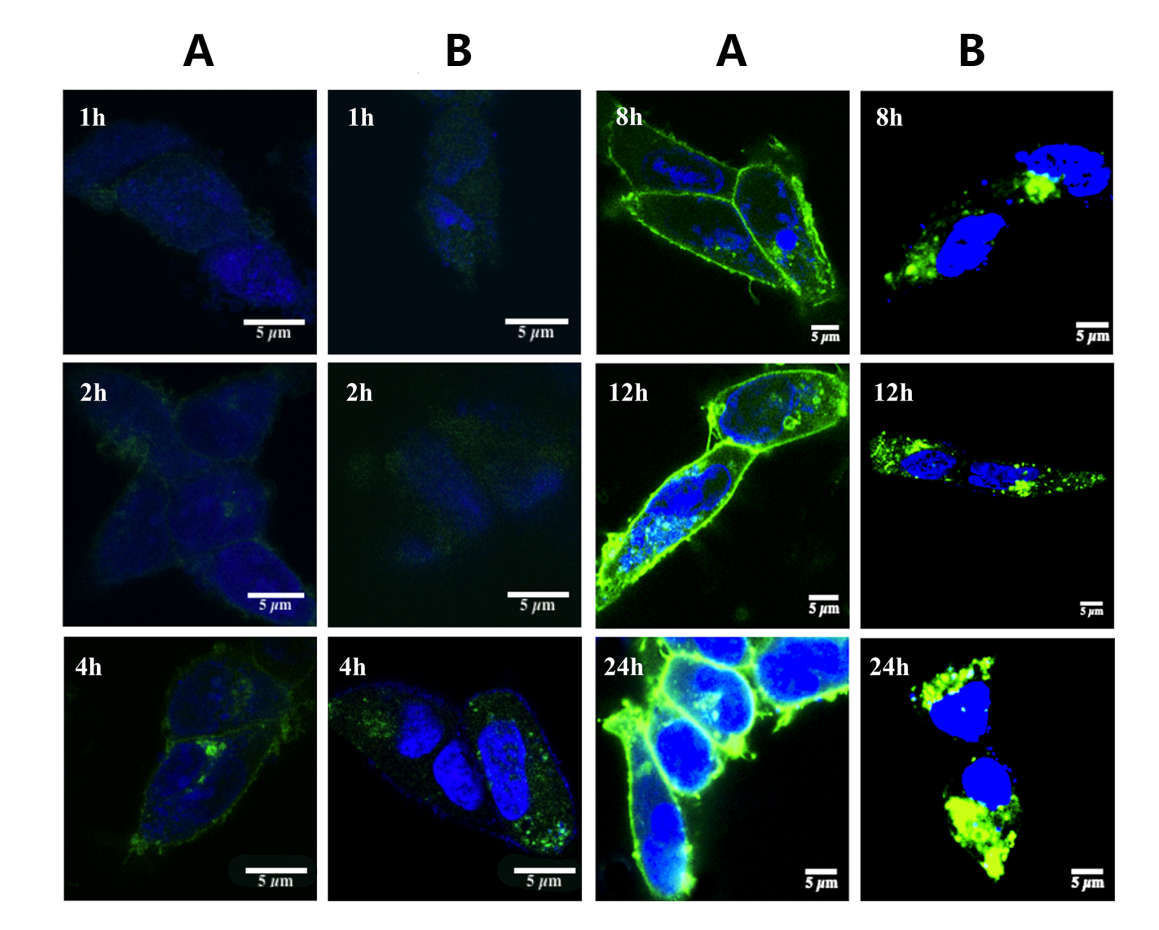


**Fig. S5 Imags of CXCR4-EGFP in living cells with and without CPZ treatment at induction times of 1 to 24 h using Confocal Microscopy.** (A) Cells treated with CPZ. (B) Cell not treated with CPZ. Green and blue represent CXCR4-EGFP and the Hoechst 33258-stained nucleus, respectively (scale bar = 5 µm).

**Movie S1: CXCR4 molecules imaged in resting cells at induction time of 4 h.** (total time 30 sec; Image size: 20µm×20µm, cyan circles represent the diffraction-limited spots).
